# Supplementary material for: Scalable and cost-effective ribonuclease-based rRNA depletion for transcriptomics
Source: Nucleic Acids Res. 2019 Dec 27;48(4):e20. doi: 10.1093/nar/gkz1169 (PMC7038938; doi:10.1093/nar/gkz1169)
Supplement: gkz1169_Supplemental_Files [file gkz1169_supplemental_files.zip › Supplementary Table Legends.pdf]

Supplementary Table Legends

Supplementary Table 1:
Sequences of oligonucleotide probes for *B. dorei*, *C. aerofaciens* and *D. longicatena*

Supplementary Table 2:
Coverage and correlation of transcriptome

Supplementary Table 3:
Primer sequences used for ssDNA generation

Supplementary Table 4:
Sequences used for adapter trimming

Supplementary Table 5:
Metadata for sequencing data

Supplementary Table 6:
Primer sequences used for library preparation

Supplementary Table 7:
Carbohydrates and SCFAs used in *B. dorei* screening experiment

Supplementary Table 8:
Annotation of up-regulated genes in *B. dorei* for SCFAs

Supplementary Table 9:
Annotation of susC/D gene pairs and PULs in *B. dorei*

Supplementary Table 10:
Probe sets designed for 5467 representative bacterial genomes in RefSeq database

Supplementary Table 11:
Cost analysis for RNase H based rRNA depletion method
